# Prepatterning in the Stem Cell Compartment

**DOI:** 10.1371/journal.pone.0010901

**Published:** 2010-05-28

**Authors:** Peter D. Tonge, Victor Olariu, Daniel Coca, Visakan Kadirkamanathan, Kelly E. Burrell, Stephen A. Billings, Peter W. Andrews

**Affiliations:** 1 Centre for Stem Cell Biology, Department of Biomedical Science, University of Sheffield, Sheffield, United Kingdom; 2 Department of Automatic Control and Systems Engineering, University of Sheffield, Sheffield, United Kingdom; University of Southern California, United States of America

## Abstract

The mechanism by which an apparently uniform population of cells can generate a heterogeneous population of differentiated derivatives is a fundamental aspect of pluripotent and multipotent stem cell behaviour. One possibility is that the environment and the differentiation cues to which the cells are exposed are not uniform. An alternative, but not mutually exclusive possibility is that the observed heterogeneity arises from the stem cells themselves through the existence of different interconvertible substates that pre-exist before the cells commit to differentiate. We have tested this hypothesis in the case of apparently homogeneous pluripotent human embryonal carcinoma (EC) stem cells, which do not follow a uniform pattern of differentiation when exposed to retinoic acid. Instead, they produce differentiated progeny that include both neuronal and non-neural phenotypes. Our results suggest that pluripotent NTERA2 stem cells oscillate between functionally distinct substates that are primed to select distinct lineages when differentiation is induced.

## Introduction

A fundamental goal of stem cell biology is to understand the mechanism(s) by which stem cells select particular pathways of differentiation. Embryonic stem (ES) cells provide a surrogate for *in vivo* development, enabling the analysis of multilineage differentiation within an *in vitro* environment. Multilineage differentiation of ES cells can be demonstrated through the simple formation of embryoid bodies (EBs), which yield cells representative of all three germ layers [1], [2]. The cell-rich, three dimensional structure of EBs increases intercellular contact, stimulating the creation of diverse cell signalling niches that support cell differentiation to a multitude of lineages. The trajectory of differentiation within EBs can also be influenced by simple parameters such as EB size, so that manipulation of EB size can be used as an effective means to bias differentiation to desired cell types [3], [4], [5]. In the absence of EB formation, ES differentiation can be directed along particular lineages by the use of defined media and/or selective passaging techniques, exemplified by numerous neural specific differentiation protocols [6], [7], [8].

However, it is not always clear whether the prescribed culture conditions actively direct differentiation to the desired cell fate or affect the outcome by promoting selective survival or proliferation of particular cell types. Cell fate choices of stem cells can be actively promoted by the manipulation of appropriate signalling pathways; for example exploitation of the Notch and SMAD signalling pathways can be used to direct ES cells to differentiate along the neural lineage [9], [10], [11]. However, identifying the relevant signalling pathway and modulating it to direct differentiation can be difficult, due to subtle differences in cell phenotypes affecting the cellular interpretation and response to particular cues. Thus, the phenotypic output of cell differentiation is not only influenced by culture conditions and signalling pathway activity but also by the phenotype of cells in the starting population. If the starting population of cells are heterogeneous, their differentiated derivatives may also be heterogeneous. This point is especially relevant when considering the demanding problem of maintaining consistent *in vitro* culture conditions.

Apparently homogeneous stem cell populations may be found to contain discrete subsets of cells that could not be initially recognised because of the absence of suitable markers. For example, human hematopoietic stem cells (HSCs) capable of multilineage hematopoietic repopulation can be purified on the basis of CD34^+^ expression and the absence of lineage markers, but this seemingly homogeneous population of stem cells was subsequently found to comprise cells that possess differing capabilities of multilineage repopulation, revealing the existence of short-term and long-term repopulating cells [12]. The heterogeneity seen within the HSC population has also been observed in leukemic stem cells, which were also once considered to be homogeneous [13]. In another example, in the intestinal crypts, stem cells were traditionally divided into a self renewing stem cell compartment and a transit amplifying compartment. However, more recent evidence suggests that the intestinal crypt stem cell compartment can be repopulated in some circumstances by cells that had apparently converted to transit amplifying cells. The repertoire of markers available for classifying stem cells is often limited, and discrimination between classes of stem cells ultimately requires functional testing [12], [13], [14].

Within the stem cell compartment there is another more subtle form of heterogeneity that is possible, whereby the cells reversibly interconvert between substates that are functionally non-equivalent while retaining the capacity for multilineage differentiation [15]. For example, mouse ES cells are capable of switching reversibly between Nanog positive and negative states, losing and gaining expression of a gene previously proposed as a key regulator of pluripotency [16]. Interconvertible Stella (+) and Stella (−) mouse ES cells have been observed and proposed to represent the switch between functionally distinct mouse ES cells and epiblast cells [17]. Human ES cells in culture may also be divided into hierarchical subsets that nevertheless can interconvert. In one study of human ES cells, we found evidence for subsets that differentially express the surface antigen SSEA3, and hypothesised that SSEA3(+) and SSEA3(−) cells can interconvert, and that the SSEA3(−) cells are closer to initiating differentiation [18]. In another study, the subsetting of human ES cells on the basis of the GCTM-2 and CD9 antigens also appeared to dissect the early stages of human ES differentiation, revealing the co-expression of pluripotency associated and lineage specific transcription factors [19]. Cells that express both sets of transcription factors may represent undifferentiated cells, lineage primed cells or transitional cell states in which the pluripotency markers have yet to be fully repressed. In the absence of functional analysis from single cell assays the nature of such cells remains elusive.

These reports are consistent with the existence of discrete interconvertible subsets of cells existing within the stem cell compartment, and that some of these subsets are closer to exiting the stem cell compartment than others. If this is the case, it might also be that different subsets are poised/primed to enter different pathways of differentiation when exposed to appropriate cues that promote differentiation [15]. Such “prepatterned” substates within the stem cell compartment could be indicated by the observations of Laslette et al (2007) in human ES cells, while previously Hu et al (1997) observed expression of lineage specific transcripts in single hematopoietic stem cells, which they suggested represents ‘lineage priming’ [20]. Thus an apparently homogeneous population of stem cells may actually comprise cells biased with respect to their differentiation potential, which are capable of generating a non-uniform differentiated population even when exposed to a uniform environment.

We have tested this hypothesis using the pluripotent human EC stem cell line, NTERA2, the differentiation of which can be easily controlled by exposure to retinoic acid (RA) [21]. Under standard culture conditions these stem cells can be maintained with minimal spontaneous differentiation, but exposure to all-trans-retinoic acid (RA) for 1–2 days is sufficient to cause almost all the cells to commit to differentiate irreversibly, after which they generate a mixed culture of neurons and other cell types. However, although prominent, the neurons only appear after 12–14 days and constitute about 1−5% of the differentiated population [21]. The nature of the other differentiated cells remains unclear although they are heterogeneous [22]; for the purpose of the present study we classified them as ‘non-neuronal’. Using this system we have now found that the individual undifferentiated EC stem cells appear to exist in interconvertible pro-neuronal or pro-non-neuronal states at, or before the point when they commit to differentiate.

## Results

### Clonogenic differentiation of NTERA2 EC cells

**Figure 1:**
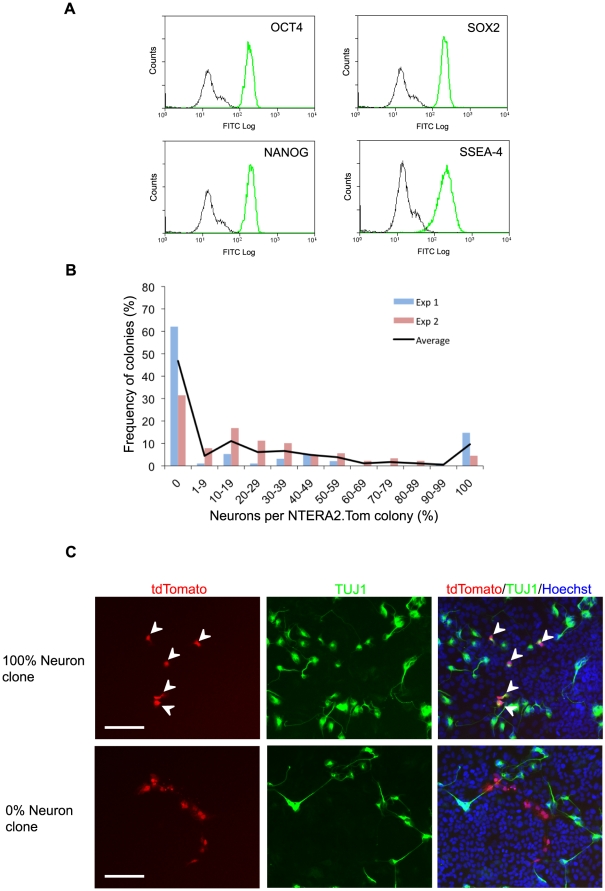
Differentiation Characteristics of Individual Stem Cells. (A) Flow cytometric analysis of undifferentiated stem cell markers in the clonal NTERA2.Tom cell line. Black line depicts P3X negative control and green depicts antigen specific expression (OCT4, SOX2, NANOG and SSEA4). (B) Graph depicting the percentage of TUJ1 positive neurons within differentiated colonies derived from individual RA treated NTERA2.Tom stem cells. (C) Images of differentiated NTERA2.Tom colonies derived from individual NTERA2.Tom cells on a background of wildtype NTERA2 cells. Cell were exposed to RA for 21 days (Green - βIII tubulin, Blue - Hoechst) White arrows depict βIII tubulin positive NTERA2.Tom neurons. Scale bar represents 50 µM.

Functional analysis of an individual cell's capacity to differentiate along the neural lineage is not amenable to clonal plating of NTERA2 cells due to an absence of neural differentiation in low density conditions, <6500 cells/cm^2^ (Tonge, P.D and Andrews, P.W. [in press] Retinoic acid directs neuronal differentiation of human pluripotent stem cell lines in a non-cell-autonomous manner. Differentiation). Thus, we generated a genetically marked clonal subline, NTERA2.Tom, which constitutively expresses the fluorescent protein, ‘tdTomato’. The NTERA2.Tom subline was clonal and used within 10 passages of cloning, to reduce the chance of genetic diversity through chromosomal drift. Cytogenetic studies revealed no heterogeneity in the fluorescent cell line with respect to the karyotype of the cells. The cells were also homogeneous with respect to the expression of stem cell associated transcription factors (OCT4, SOX2 and NANOG) and the surface antigen SSEA4 (Figure 1A).

Single NTERA2.Tom cells were seeded with a background of wild type NTERA2 cells at a density that permits neuronal differentiation (15000 cells/cm^2^), and induced to differentiate by the addition of RA. After three weeks the progeny derived from individual NTERA2.Tom cells were assessed for a neuronal or non-neuronal phenotype on the basis of β-III tubulin expression and cell morphology. As the NTERA2.Tom subline appears phenotypically homogeneous it might be expected that each RA treated cell would yield differentiated colonies containing similar proportions of neurons. However, the differentiated colonies derived from single NTERA2.Tom cells did not conform to a single stereotype, and the percentage of neurons in each colony ranged between 0 to 100% (Figure 1B). The presence of colonies composed entirely of neurons, or containing no neurons (Figure 1B, C) suggests that the eventual phenotype of the differentiated cells is a consequence of a lineage decision made at the point of induction to differentiate.

Although the NTERA2.Tom cell line was clonal, we confirmed the absence of parallel co-existing variant cells that stably possess differing propensities to yield neurons upon RA exposure, by recloning the NTERA2.Tom cells and testing several subclones for neuronal differentiation. Each subclone gave similar proportions of neurons following differentiation (Figure S1), indicating that the appearance of distinct neuronal and non-neuronal differentiated clones was not due to distinct sublines of cells within the NTERA2.Tom cell line.

On the premise that undifferentiated NTERA2 stem cells are biased with respect to neuronal differentiation, and since lineage biased cells were not evident upon subcloning, we hypothesized that the lineage biased cell substates are interconvertible. To test whether the undifferentiated stem cell population consists of such interconvertible substates we carried out an experiment in which the addition of RA to cultures of plated cells was delayed for variable lengths of time, allowing undifferentiated stem cells to divide at least once before initiation of differentiation. In this case, if the lineage biased substates are readily interconvertible, it was anticipated that allowing the NTERA2.Tom cells to divide prior to RA exposure would increase the number of mixed colonies at the expense of homogeneous neural or non-neural colonies, since the initial single EC cell would give rise to 2, 3 or 4 such cells, which could adopt either the pro-neural or pro-non-neural state, before being exposed to RA.

**Figure 2:**
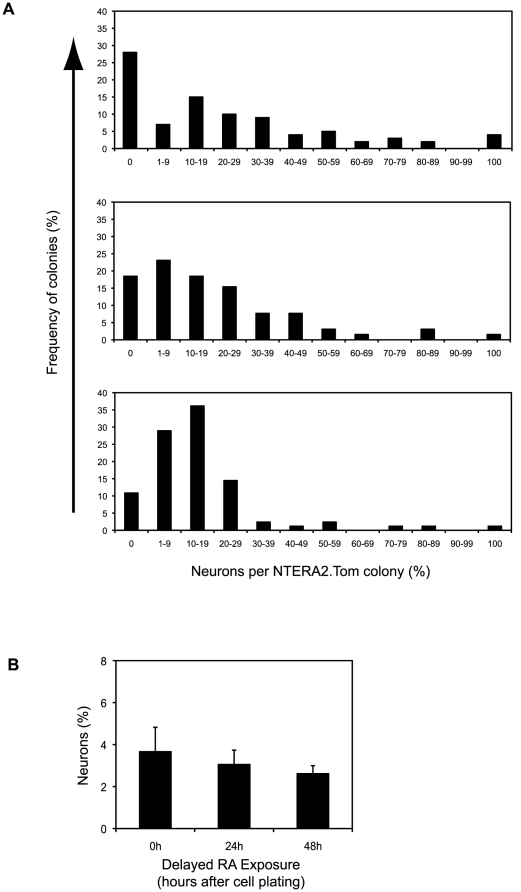
Delayed Initiation of Differentiation Alters Phenotype of Single Cell Derived Differentiated Colonies. (A) Analysis of neuronal yield within differentiated colonies derived from individual RA treated NTERA2.Tom stem cells. NTERA2.Tom cells were plated on a background of wildtype NTERA2 cells and exposed to RA at varying time points after cell plating (O, 24, 48 and 72 hours). >100 colonies were analysed for each time point. Data is derived from a single experiment and representative of three independent experiments. (B) Neuronal yield of RA treated NTERA2 cells on a population basis. Cells were exposed to RA at either 0, 24 or 48 hours after cell plating. Percentage of neurons calculated after 21 days RA exposure.

When RA induction of differentiation was postponed by 24 h or 48 h after plating of cells, the number of mixed phenotype colonies did increase at the expense of homogeneous neuronal and non-neural colonies (Figure 2A). Postponing RA treatment by 72 h further increased the number of mixed colonies with less than five percent of differentiated colonies lacking neurons when RA addition had been postponed 72 hours, although in this case the NTERA2.Tom colonies were too large and compact to accurately quantify percentage of neurons (Figure S2). As the delay between cell plating and RA addition increased, the percentage of neurons in each colony converged to the figure that represents average neuronal yield of a population of RA treated NTERA2 stem cells (4−6%). Although delaying RA exposure altered the neuronal content of single cell derived differentiated colonies the percentage yield of neurons in the overall cell culture remained unchanged (Figure 2B).

Commitment to differentiate is not instantaneous but occurs over a period of 24–48 hours (Figure S3A). In non-synchronous cultures about 50% of the cells divide in the first 24 h (Figure S3B) so that a proportion of colonies would arise from the differentiation of more than one stem cell, which might be in alternate pre-patterned states at the time of commitment. That the inferred pro-neuronal and pro-non-neuronal substates can interconvert explains why there are also some mixed colonies even when RA induction is initiated at the time of seeding the NTERA2.Tom cells.

### Experimental Data Modelling Results

**Figure 3:**
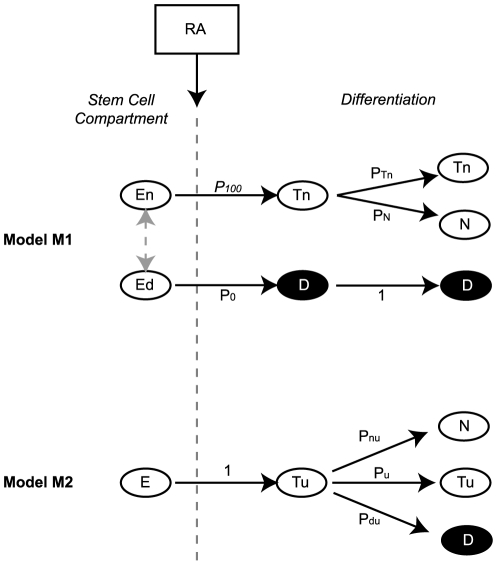
Models of Lineage Selection During Cell Differentiation. (**A**) Model M_1_ schematic describing the committed scenario where NTERA2 cells make the neural/non-neural lineage choice upon induction to differentiate. Grey arrow denotes a choice between two theoretical substates. En = undifferentiated stem cell with a selected neural fate, Ed = undifferentiated stem cell with a selected non-neuronal fate, Tn = neuronal progenitors, D = non-neuronal progenitors and non-neuronal differentiated cells, N = neuron, p_100_ = probability to choose neuronal fate, p_0_ = probability to choose non-neuronal fate, p_Tn_ = probability of neuronal progenitors to proliferate, p_N_ = probability to differentiate into neurons. (**B**) Model M_2_ schematic describing the uncommitted scenario when RA was added at time of cell plating. E = undifferentiated stem cell with unselected fate, N = neuron, D = non-neuron, Tu = uncommitted progenitor with unselected fate, p_nu_ = probability to differentiate into neurons, p_u_ = probability of progenitor Tu to proliferate., p_du_ = probability to differentiate into non-neurons.

To consider our interpretation of these results further, we carried out a mathematical simulation of stem cell differentiation, considering two opposing models by which undifferentiated NTERA2 stem cells can give rise to a population composed of two types of differentiated cells, neuronal or non-neuronal (Figure 3). The first model (*M*_1_) assumes that commitment to differentiation and lineage selection occurs simultaneously in response to RA, whereas the second model (*M*_2_) assumes that commitment to differentiation occurs first, and that lineage selection occurs subsequently after several cell divisions.

In *M*_1_, a fraction, *p*_100_, of undifferentiated cells, En, is biased to the neuronal lineage and eventually only yields neuronal progeny, whereas the remaining fraction, Eu, of undifferentiated cells, *p*_0_ (*p*_100_+*p*_0_ = 1), is non-neuronal biased and only gives rise to non-neuronal cells. In standard culture conditions, in the absence of differentiation cues, the undifferentiated cells are assumed to interconvert freely between the neuronal and non-neuronal biased states, so that following cell division the fraction of neuronal biased cells remains constant. We further assume that after exposure to RA, the cells that have selected a neuronal fate may continue dividing as committed neuronal precursors, Tn, and at each cell division these may generate similar proliferating precursors with a probability *p*_Tn_, or differentiate into terminal non-dividing neurons, N, with a probability *p*_N_. For simplicity we assume that the cells selecting a non-neuronal fate, D, continue dividing. By contrast, in *M*_2_ the undifferentiated cells exist in a single, unbiased state, E, and after RA treatment only yield uncommitted precursors, Tu, that continue dividing. At each cell division, these uncommitted precursors may yield similar uncommitted precursors with a probability *p*_u,_, or proliferating precursors committed to a non-neuronal fate with a probability *p*_du_, or terminally differentiated neurons with a probability *p*_nu_.

**Figure 4:**
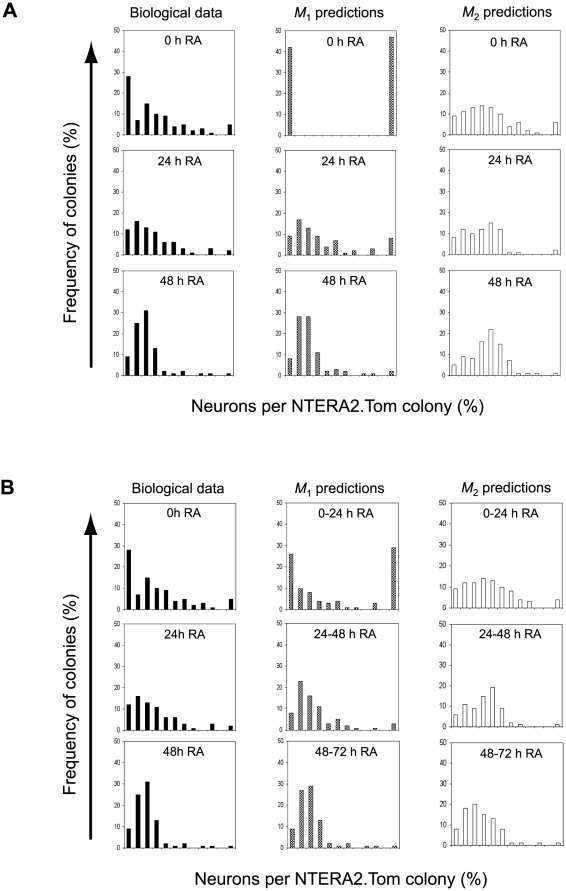
Correlation of Biological and Model Data. (A) The first column illustrates the frequency distribution of colonies with regards to percentage of neurons obtained from biological data. The second and third columns show the frequency distribution of colonies predicted by models *M*_1_ and *M*_2_ respectively. Data is representative of when RA is added at either 0, 24 or 48 hours post cell plating. (B) The first column illustrates the frequency distribution of colonies with regards to percentage of neurons obtained from biological data. The second and third columns show the frequency distribution of colonies predicted by model *M*_1_ and model *M*_2_ respectively. The model data is obtained from merged results for the cases where RA was added at 0 and 24 hours, 24 and 48 hours, or 48 and 72 hours.

To decide which model better describes the clonal differentiation of NTERA2.Tom EC cells, we compared the probability density functions of the percentage number of neurons **n** in a colony {*f*_0_(**n**), *f*_24_(**n**), *f*_48_(**n**)}, (obtained from the biological data shown in Figure 2), for the three cases in which RA was added at 0 hours, 24 hours and 48 hours after initial seeding), with the probability density functions of **n** {*m*_1,0_(**n**|*θ*_1_), *m*_1,24_(**n**|*θ*_1_), *m*_1,48_(**n**|*θ*_1_)} and {*m*_2,0_(**n**|*θ*_2_), *m*_2,24_(**n**|*θ*_2_), *m*_2,48_(**n**|*θ*_2_)} generated through Monte Carlo simulations of models *M*_1_ and *M*_2_ respectively (Figure 4A). The parameter vectors associated with each model 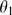 = {*p*_0_, *p*_100_ = 1- *p*_0_, *p*_Tn_, *p*_N_ = 1- *p*_Tn_} and 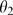 = {*p*_nu_, *p*_u_, *p*_du_ = 1- *p*_nu_ - *p*_u_} were estimated from data using a grid search approach. As a measure of closeness or goodness-of-fit of {*f*_0_(**n**), *f*_24_(**n**), *f*_48_(**n**)} with respect to {*m*_1,0_(**n**|*θ*_1_), *m*_1,24_(**n**|*θ*_1_), *m*_1,48_(**n**|*θ*_1_)} and with {*m*_2,0_(**n**|*θ*_2_), *m*_2,24_(**n**|*θ*_2_), *m*_2,48_(**n**|*θ*_2_)} we used the Kullback–Leibler (KL) divergence criterion [23], [24]. To demonstrate the applicability of this approach to distinguish between the two hypothesized models, we first carried out a numerical simulation study using data generated through Monte Carlo simulations of *M*_1_ and *M*_2_ with a known set of parameters (Figure S4).

For the models estimated from the experimental observations, model *M*_1_ outperformed *M*_2_ when RA was delayed 24 or 48 hours (Figure 4A and Table 1). However, in the case when RA exposure commenced upon cell plating, neither model performed well. Essentially, model *M*_1_ predicts that only two types of colonies may form: colonies that contain 100% neurons or 0% neurons. This is a direct consequence of the simplifying assumption that single NTERA2.Tom cells commit to differentiate immediately upon RA exposure at 0 hours, prior to any cell division, i.e. that the cells are fully synchronised. In reality this is not the case: only about 50% of cells divide within the first 24 hours (Figure S3) with the first division for most cells occurring at any time during the first 48 hours after seeding. Also, whereas exposure to RA for ≥3 days induced over 99% of NTERA2 stem cells to differentiate, only 50% of cells commit to differentiate when exposed to RA for 24 hours (Figure S3A). If Model *M*_1_ is correct and lineage biased (interconvertible) substates exist, an undifferentiated stem cell could divide and the two progeny acquire discrete substates during the period immediately following exposure to RA. If we consider that some RA exposed NTERA2.Tom cells divide before they commit to differentiate, then the biological 0 hours RA experiment is actually represented by a mix of the 0 hour and 24 hour RA model data. To investigate this further, for each model we computed density functions using a mixture of simulated data (0 h and 24 h, 24 h and 48 h etc). These were compared to the biological data (Figure 4B). As it can be seen in Table 2, the relative entropies between the model-generated mixed density functions and density functions estimated from experimental data were reduced significantly, particularly for Model *M*_1_. These results demonstrate that out of the two proposed models of lineage choice during differentiation, the committed stem cell model *M*_1_ best predicts the distribution of the percentage number of neurons in colonies derived from individual stem cells.

**Table 1:** Kullback-Leibler Divergences Between Biological and Model Generated Data Sets.

RA added after plating	Model *M*_1_		Model *M*_2_	
	*KL*_1,*j*_	*I*_1_ = *KL*_1,0+_ *KL*_1,24+_ *KL*_1,48_	*KL*_2,*j*_	*I*_2_ = *KL*_2,0+_ *KL*_2,24+_ *KL*_2,48_
0 hours	1.174	1.827	0.943	3.027
24 hours	0.372		0.937	
48 hours	0.281		1.162	

*KL* divergences and overall cost function *I* between density functions estimated from biological and model generated data.

**Table 2:** Kullback-Leibler Divergences Between Biological and Merged Model Generated Data Sets.

RA added after plating	Model *M*_1_		Model *M*_2_	
	*KL*_1,*j*_	*I*_1_ = *KL*_1,0+_ *KL*_1,24+_ *KL*_1,48_	*KL*_2,*j*_	*I*_2_ = *KL*_2,0+_ *KL*_2,24+_ *KL*_2,48_
0 hours	0.283	0.446	0.492	1.487
24 hours	0.141		0.516	
48 hours	0.042		0.479	

*KL* divergences and overall cost function *I* between density functions estimated from biological data and a mixture of model simulated data.

## Discussion

Much of the heterogeneity seen in populations of stem cells might be compatible with transcriptional ‘noise’ that does not have functional significance. However, our present results indicate that individual pluripotent NTERA2 human EC cells do exist in functionally distinct substates that are primed to adopt specific eventual fates before they commit to differentiate. Further, these ‘pre-patterned’ substates are interconvertible while the cells still retain a stem cell phenotype. Our interpretation of the experimental data was supported by a Monte Carlo simulation of the two opposing models, namely that selection of eventual neuronal or non-neuronal fates depends upon events occurring before or co-incident with induction of differentiation, rather than following cell commitment to differentiate.

The possibility that heterogeneity within stem cell populations may have functional consequences for stem cell self-renewal, commitment to differentiation, and lineage choice upon differentiation is an idea that has been developing for a number of years [15]. In this context, heterogeneity does not refer to mixtures of cells that become evident as better tools evolve for distinguishing distinct cell types within a population, but rather represents the existence of multiple, interconvertible cell states that together constitute a stem cell compartment. Such heterogeneity became apparent when single cell PCR analyses of hematopoietic stem cells revealed ‘lineage priming’, the expression of lineage related genes in individual cells that still expressed a stem cell phenotype, [20], [25]. Lineage priming suggests that the stem cells activate specific genes associated with their differentiated progeny before they commit to differentiate. Similar heterogeneity within the stem cell compartment occupied by mouse ES cells and corresponding cells in the mouse conceptus may also be pertinent to fate decisions in early embryogenesis. Expression of the key regulatory gene, *Nanog*, fluctuates not only in mouse ES cells but also in the inner cell mass [16]. High levels of *Nanog* expression appear to stabilise the undifferentiated state of these pluripotent cells, whereas loss of *Nanog* expression appears to represent a step towards eventual differentiation; although a lack of *Nanog* expression is compatible with the cells retaining an undifferentiated pluripotent phenotype, such cells are more unstable and prone to differentiate. Co-expression in early embryonic cells of genes associated with the stem state, such as *Oct4*, with genes associated with specific fates, such as *Cdx2* (trophectoderm) or *Gata6*, or *Pdgfrα* (extraembryonic endoderm) have been observed [26]. It has been argued that the pluripotent ES cell state represents a metastable ground state in which a fluid transcriptome allows for its multilineage potential [27].

A theoretical basis for this phenomenon has been provided by a model of a myeloid stem cell, based upon a simple gene regulatory network of the two transcription factors associated with monocyte and erythroid differentiation, Pu.1 and GATA1 [28], [29]. This model predicts not only the existence of two stable states, mathematically described as attractors [30], corresponding to the differentiated monocytes and erythrocytes, but also another metastable attractor corresponding to the uncommitted stem cells, in which both transcription factors are expressed at low level. Recently, it was reported that fluctuations in the levels of Pu.1 and GATA1 within such a metastable stem cell attractor biases the fate decisions of the stem cells when they commit to differentiate, but this was not confirmed at the single cell level [31]. Gene regulatory networks involving Oct4, Sox2 and Nanog may similarly define the pluripotent state of ES cells, by interacting with and suppressing other networks that specify differentiation along particular lineages [27].

An alternative to a strict gene regulatory network mechanism of lineage priming or pre-patterning, would be a mechanism involving epigenetic regulation. It has been recognised for many years that pluripotent stem cells have a more open chromatin structure than their differentiated derivatives [32], perhaps permitting ‘leaky’ gene expression. Much more recently it was reported that in ES cells the histones associated with the chromatin of various developmentally regulated genes, notably transcription factors, show bivalent repressive and activation marks [33], [34]. Such chromatin modifications may play a key role in lineage specification [35] and a model involving “histone modification pulsing” has been suggested as a mechanism whereby individual pluripotent cells may acquire differential fates [36]. Whether interconvertible lineage primed, or pre-patterned substates of pluripotent stem cells are maintained by dynamic gene regulatory networks, or by epigenetic factors, or a combination of both, the observation that stem cells can oscillate between such states should be incorporated into our understanding of cell differentiation during embryogenesis. It may also be pertinent to understanding pathological states of disordered cell differentiation as in cancer.

## Methods

### Cell culture

Undifferentiated NTERA2 cl.D1 EC cells were grown in DMEM growth medium (Dulbecco's Modified Eagle's Medium, GIBCO) supplemented with 10% fetal bovine serum (Hyclone), and maintained at 37°C in a humidified atmosphere of 10% CO_2_ [21]

### Generation of NTERA2.Tom fluorescent cell line

Stable cell lines that constitutively expressed the fluorescent protein tdTomato were generated by transfection of undifferentiated NTERA2 clD1 EC cells. Cells were electroporated with 15μg of plasmid DNA that contained a tdTomato-ires-Pac cassette under the control of the CAG promoter. Puromycin resistant, tdTomato fluorescent colonies were clonally picked and expanded. An NTERA2.Tom clonal subline was expanded and assessed to ensure that tdTomato expression was not diminished upon RA induced cell differentiation.

### Neuronal differentiation

RA mediated differentiation [21] was performed by plating NTERA2 stem cells at 15000 cells/cm^2^ in standard growth media (10% FBS in DMEM) supplemented with 10^-5^M all-*trans*-retinoic acid (Sigma). The all-*trans*-retinoic acid (RA) was stored as a DMSO stock solution of 10^−2^M, with RA supplemented growth media replenished every seven days.

Confluent NTERA2.Tom and wildtype NTERA2 stem cells were trypsinised and ensured that ≥99% of NTERA2.Tom cells were single cells. The NTERA2 stem cells were mixed at a ratio of one NTERA2.Tom cell per 2000 wildtype cells and the cell suspension plated at a density amenable to neuronal differentiation (15000 cells/cm^2^), in the presence of 10^−5^M RA. Four hours after plating the cells in 96 well plates, each well was checked by fluorescence microscopy to ensure that all fluorescent (NTERA2.Tom) cells were single cells and that no two fluorescent cells were closely situated to one another.

### Immunofluorescence and Flow Cytometry

All cell staining for βIII tubulin was performed after 21 days of RA induced differentiation. In situ immunofluorescence was performed after fixation in 4% paraformaldehyde (PFA) fixation of cells, with the use of the monoclonal antibody TUJ1 (Covance) in conjunction with Alexa Fluor 488 secondary antibody (Molecular probes).

Flow cytometry analysis was performed on cell suspensions as previously described (Fox, Gokhale et al. 2008). For intracellular staining, cells were PFA fixed and permeabilised with 0.1% triton X100. Cell suspensions were stained with monoclonal antibodies to SOX2 (R&D Systems, MAB1018), OCT4 (Santa Cruz Sc-9081), NANOG (R&D systems, AF1997), TUJ1 (Covance) and SSEA4, diluted in PBS containing 5% goat serum. Antibody to SSEA4 was produced from hybridoma MC813-70 [37]. Cell suspensions were analysed using a CyAN (DakoCytomation) with Summit software. Non-specific fluorescence staining and autofluorescence was determined by P3X, an antibody obtained from the myeloma cell line P3X63Ag8 [38] which does not recognize any known epitope in the cells.

### Parameter estimation

The parameter vectors 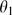 = {*p*_0_, *p*_100_ = 1- *p*_0_, *p*_Tn_, *p*_N_ = 1- *p*_Tn_} and 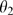 = {*p*_nu_, *p*_u_, *p*_du_ = 1- *p*_nu_ - *p*_u_} of the two competing models *M*_1_ and *M*_2_ were estimated from data using a grid search approach. As a measure of closeness or goodness-of-fit between the density functions *F*(**n**)* = *{*f*_0_(**n**), *f*_24_(**n**), *f*_48_(**n**)} estimated from experimental data for the three cases in which RA was added at 0 hours, 24 hours and 48 hours (Figure 4) and the model-generated density functions *M*_1_(**n**|*θ*_1_) = {*m*_1,0_(**n**|*θ*_1_), *m*_1,24_(**n**|*θ*_1_), *m*_1,48_(**n**|*θ*_1_)} and *M*_2_(**n**|*θ*_1_) = {*m*_2,0_(**n**|*θ*_2_), *m*_2,24_(**n**|*θ*_2_), *m*_2,48_(**n**|*θ*_2_)}, we used the Kullback–Leibler (KL) divergence KL*_i,j_* = KL(*f_j_* (**n**);*m_i,j_* (**n**|*θ*_1_)), (*j* = 0,24,48; *i* = 1,2) which is a non-commutative measure of the difference between two probability distributions [23]. Specifically, the optimization goal was to minimize the following cost function
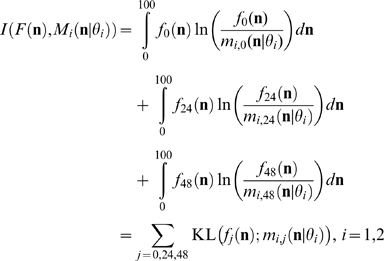
(1)


For each model only two parameters have to be estimated. For *M*_1_ the parameters *p*_0_ and *p*_Tn_ were estimated using a standard grid search approach. For *M*_2_, the parameters *p*_nu_ and *p*_u_ were estimated using a restricted grid search, which only considered feasible values of the parameters so that the constraint *p*_nu_+*p*_u_+*p*_du_ = 1 was satisfied.

Each parameter was allowed to take 101 possible values [0, 1/100, 2/100,…,1], with no restrictions for *M*_1_ and with the restriction *p*_nu_+*p*_u_ ≤1 for model *M*_2_. For each parameter set, 100 Monte Carlo iterations were carried out. The resulting data were used to derive the distribution functions associated with each model, which in turn were used to evaluate the cost function in equation (1). The density functions in equation (1) were computed using kernel methods. The reasons for adopting grid search strategies for parameter optimization are that these techniques are not susceptible to the local optimum problem and for this particular application are not computationally expensive [39].

Tables 1 and 2 summarize the performance of each model relative to the experimental data. According to the minimum discrimination information principle, the model with a smaller relative entropy is the better one [40]. In our case, clearly model *M*_1_ outperforms *M*_2_.

Because the models have the same number of parameters, there was no need to use more sophisticated information or theoretical criteria such as the Akaike's information criterion (AIC) [41], [42] or the improved versions of AIC which have been proposed and applied to Monte Carlo models by Bozdogan [43], [44], since the term which penalizes complex models would be redundant [45].

The proposed model estimation and selection approach was also evaluated using two sets of density functions *D*_1_(**n**|*θ*_1_) = {*m*_1,0_(**n**|*θ*_1_), *m*_1,24_(**n**|*θ*_1_), *m*_1,48_(**n**|*θ*_1_)} and *D*_2_(**n**|*θ*_2_) = {*m*_2,0_(**n**|*θ*_2_), *m*_2,24_(**n**|*θ*_2_), *m*_2,48_(**n**|*θ*_2_)} generated using Monte Carlo simulations of the two models *M*_1_ and *M*_2_. The model parameters were 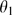 = {*p*_0_ = 0.65, *p*_100_ = 1- *p*_0_ = 0.35, *p*_Tn_ = 0.45, *p_N_* = 1- *p*_Tn_ = 0.55} for model *M*_1_ and 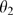 = {*p*_nu_ = 0.3, *p*_u_ = 0.4, p_du_ = 1- *p*_nu_ - *p*_u_ = 0.3} for model *M*_2_. 100 single cells were assumed to divide and differentiate according to the two alternative models *M*_1_ and *M*_2_ assuming that a retinoic acid dose was added from the start and 24 and 48 hours later. The number of cell divisions (*N*_d_) was set to 6 for both models.

Two sets of parameters were estimated for each model, one set fitted using data generated by the model itself (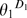 for *M*_1_ and 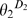 *M*_2_), the other fitted on data generated by the competing model (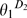 for *M*_1_ and 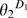 *M*_2_).

Table S1 shows in each case the best set of estimated parameters, the root mean square error (*RMSE*) between the true and estimated parameters
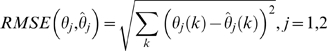
and the values of the cost function in equation (1) computed using the original density functions *D*_1_, *D*_2_ and the model predicted density functions *M*_1_ and *M*_2_.

We found that there is a good agreement between estimated and true parameters of models *M*_1_ and *M*_2,_ which were estimated based on the ‘correct’ sets of synthetic density functions *D*_1_ and *D*_2_ respectively. In each case the estimated models are able to predict well the original data sets. However, when parameters of *M*_1_ are fitted using the *D*_2_ data set and parameters of *M*_2_ are fitted using the *D*_1_ data set, the estimated models perform significantly worse. These results demonstrate that the proposed approach can recover from data the original parameters for each model and that the two models lead to distinct outcomes. Following parameter optimization, none of the models can predict satisfactory the data generated by the other.

## Supporting Information

Figure S1 Sub-clones of the NTERA2.Tom cell line assayed for the percentage of βIII tubulin positive neurons yielded upon RA mediated differentiation.
(1.13 MB EPS)

Figure S2 (A) Differentiated colony derived from a single NTERA2.Tom cell plated on a background of wildtype NTERA2 cells. Cells were exposed to RA 72 hours after plating and stained for TUJ1 after 21 days of RA exposure. (B) Population based analysis of neuronal yield. Cells were plated and exposed to RA 0, 24 or 48 hours after plating.
(4.80 MB EPS)

Figure S3 (A) NTERA2 cells were exposed to RA (10uM) for different periods of time (24 hrs, 3 days, 6 days and continuous exposure) and then clonal plated in the absence of RA. The graph depicts the number of undifferentiated colonies produced ten days after clonal plating. Data is representative of a three independent experiments expressed as a mean value. (B) Time between cell plating and first cell division of individual cells, in RA containing media. Data is derived from time-lapse microscopy of cells.
(2.18 MB EPS)

Figure S4 (A) Distribution of colonies with regards to percentage of neurons from synthetic data for M1 (Column 1), the distributions of colonies with regards to percentage of neurons estimated by M1 (Column 2) and the distributions of colonies with regards to percentage of neurons estimated by M2 (Column 3) for all 3 cases: Retinoic Acid (RA) added at 0 hours, 24 hours and 48 hours. (B) Distributions of colonies with regards to percentage of neurons from Synthetic data for M2 (Column 1), the distributions of colonies with regards to percentage of neurons estimated by M2 (Column 2) and the distributions of colonies with regards to percentage of neurons estimated by M1 (Column 3) for all 3 cases: Retinoic Acid (RA) added at 0 hours, 24 hours and 48 hours.
(2.75 MB EPS)

Table S1 Original and estimated M1 and M2 parameters based on the D1 and D2 data sets, the corresponding parameter estimation errors and the values of the cost function computed for density functions generated by original and estimated models.
(0.61 MB EPS)
